# Voluntary suppression of neck reflexes during passive head-on-trunk rotations: reflex gain control versus proprioceptive feedback

**DOI:** 10.1152/jn.00297.2021

**Published:** 2021-12-15

**Authors:** Dimitri Anastasopoulos, Lysandros Anastasopoulos, Thomas Mergner

**Affiliations:** ^1^Department of Neurorehabilitation, Zurzachcare, Bad Zurzach, Switzerland; ^2^Department of Physiology, University of Athens, Athens, Greece; ^3^Speedgoat GmbH, Liebefeld, Switzerland; ^4^Department of Neurology, University of Freiburg, Freiburg, Germany

**Keywords:** dynamic neuromechanical model, motor control, neck reflexes, passive movements

## Abstract

Normal subjects can completely eliminate resistance upon imposed head-on-trunk rotations when they are asked to relax. It is not, however, clear how neck reflexes to stretch can be voluntarily suppressed. Reflexive responses might be modified by adjusting the gain of the reflex loop through descending control. Theoretically, necessary corrections upon interfering disturbances during coordinated motor performance requiring the interplay of relaxation/activation may be missing if muscle relaxation is taking place exclusively by this mechanism. It has been alternatively proposed that sensory information from the periphery may be allowed to “neutralize” neck reflexes if it is fed back with opposite sign to the structures driving the reflexes. Six healthy subjects were asked to relax while subjected to head-on-trunk rotations generated by a head motor. After any initial resistance had completely subsided, the head was unexpectedly exposed to “ramp-and-hold” perturbations of up to 2° amplitude and 0.7 s duration. Resistance to stretch consistently reappeared thereupon, suggesting that stretch reflex gain had not been set to zero during the previously achieved complete relaxation. Resistance to perturbations under these circumstances was compared with the forces generated when the same ramp-and-hold displacements were delivered unpredictably to the head held stationary. A quantitative model of neck proprioceptive reflexes suppression has been thus constructed. Gain scheduling or “motor set” cannot sufficiently account for the voluntary reflex suppression during slow passive head rotations. Instead, we propose as underlying mechanism, the “neutralization” of the controlling servo by means of continuous feedback tracking displacement and force signals from the periphery.

**NEW & NOTEWORTHY** Head stabilizing neck reflexes can be voluntarily suppressed or activated depending on the task at hand. By applying brief perturbations unexpectedly, both during passive head-on-trunk movements and at rest, we investigated the mechanism of voluntary suppression of resistance to stretch. A physiologically plausible, neuromechanical model of voluntary/reflexive interactions was constructed favoring feedback over reflex gain adjustments. Accordingly, muscle relaxation during imposed head movements is based on sensory feedback similarly to muscle contractions during purposeful movements.

## Graphical abstract

**Figure:**
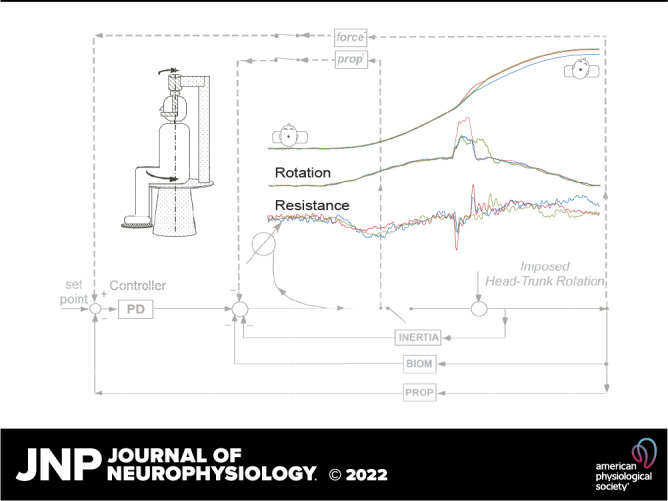

## INTRODUCTION

Neck muscles can be reflexively activated and contribute to head stabilization against external disturbances. Head motion in space, detected by vestibular sensors, is counteracted by the vestibulocollic reflex, whereas head-to-trunk motion is opposed by the proprioceptive cervicocollic reflex (1). These reflexes can be activated or suppressed voluntarily because, depending on the task, the head may be required to be stabilized at times “in space” (i.e., in a certain direction, e.g., in boxing) or “to the trunk” (e.g., in jogging; 2).

Clinicians routinely assess neuromotor status in several motor disorders (such as Parkinson’s disease or cervical dystonia) by rotating limb joints and the head of the patient who has been asked to relax. It is, however, not clear how normal subjects manage to relax their neck muscles and cancel neck reflexes during passive head rotations when they are asked to. Modifiable reflexive responses of limb muscles to stretch, assessed mainly from electromyogram (EMG) recordings, have been demonstrated by numerous studies in the last 60 years (3–6). It has been concluded that mainly the “long-latency” components of stretch responses are task dependent; they are modulated by the subject’s intent, the behavioral context, and are subjected to environmental constraints (7, 8). It has been recently suggested that the long-latency reflexes and the voluntary motor system are intimately linked because they both rely on feedback control and engage similar neural circuitries including the primary motor cortex (9). Indeed, the primary motor cortex and the supplementary motor area are equally activated when joint rotations at the elbow and wrist are executed either actively through voluntary muscle contraction or passively when asked to voluntarily relax (10, 11). Descending motor commands have been thought to change long and even short-latency reflex transmission, supposedly by reducing the gain of the reflex pathway (“gain scheduling”; 12, 13).

In previous investigations (14, 15), we asked normal subjects and patients with movement disorders not to resist imposed head-to-trunk deflections in yaw and evaluated the resistance exerted on the slowly rotating head holder. After an initial rise of resistance, normal subjects were capable of completely eliminating any opposing force to the rotating device in the course of the passive movement. Based on these investigations, we proposed that the head-neck reflexes to stretch are thereby voluntarily cancelled by a “mirror” proprioceptive loop conveying information concerning head-trunk angle change, that is, relaxation was thought to rely on feedback control rather than to be affected by stretch reflex gain adjustments. Notably, Corna et al. (16) had earlier described short-latency EMG responses in neck muscles when an abrupt force was exerted on the head and the subjects were asked to relax. Under these circumstances, subjects not only suppressed stretch reflexes in the lengthening muscles but at the same time they also activated at short-latency muscles assisting the head in following the direction of the imposed force. Clearly, suppression by gating the gain of neck reflexes in lengthening, relaxed muscles was not the exclusive source of resistance reductions. Apart from the estimation of the neuromuscular suppression/activation latencies, the study of Corna et al. (16) did not provide information about the mechanism and quantitative aspects of the suppressive process.

We now exposed the head unexpectedly to “ramp-and-hold” displacement perturbations (1–2°, duration between 0.3 and 0.7 s) in midcourse of the deployment of a slow, externally generated, “passive” head-to-trunk rotation after any resistance from the head-neck system had completely subsided and measured the generated forces (i.e., torque) in response to these perturbations. At this particular time, the suppressive mechanism is presumably at work and stretch reflexes of the lengthening, “giving way” muscles are supposedly inactivated. Any evoked resistance upon the perturbations at this instance would offer direct evidence that the relaxation process involves sensory feedback. In contrast, no reflex response thereupon would be expected, if stretch reflex gain adjustments (i.e., gain attenuated to ∼0) were exclusively responsible for the immediately previously achieved complete relaxation. Exactly the same brief displacement perturbations were also applied with the head held stationary, that is, when the suppressive mechanism is still inoperative. The components and parameters of the hypothesized suppression process were thus estimated by the way of direct comparisons with predictions of the previously proposed model.

## METHODS

### Subjects

Six healthy university students (3 males, 21–22 yr) participated who had not suffered any neuromuscular disease in the past and had not been previously exposed to laboratory measurements. All subjects provided written informed consent, and the protocol was approved by the Human Research Ethics Committee at the University of Athens in accordance with the WMA Declaration of Helsinki (ethics ID 119).

### Apparatus

**Figure 1.**
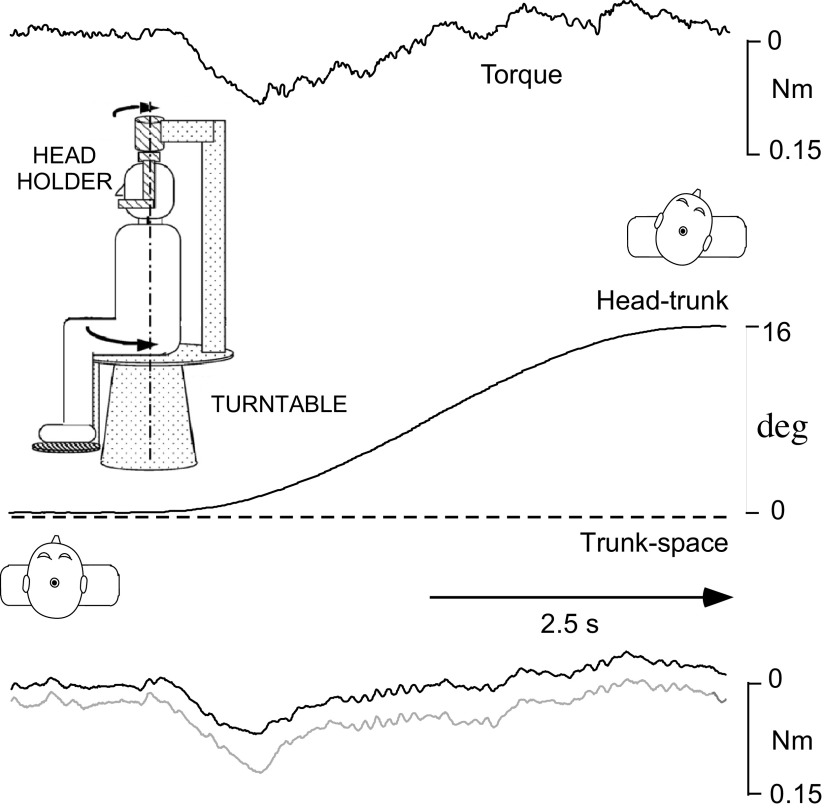
Representative raw torque response to a head-only smoothed trapezoidal, 5 s externally generated rotation to the right (*top* record) and the average response of the 6 subjects (*bottom* records, bold line) minus 1 SD (thin gray line). The trunk remains stationary. Subjects have been asked not to resist head movements driven by the head motor. Negligible force is exerted to the head holder at rest. Resistive torque develops counter (downward) to head-on-trunk deflection with an initial relatively sharp rise phase. Resistance begins to decline within several hundreds of ms after stimulus onset. Within approximately 2 s after the stimulus onset, resistive torque has completely subsided and dropped to 0; the head is being moved “passively” as subjects are trying to comply with the instruction to relax. *Insets* show the experimental arrangement and the head-on-trunk positions at the beginning and end of the rotation.

Subjects were seated on a Bárány chair in the center of a cylindrical screen with a radius of 0.8 m. A bite-board, specifically molded from each individual’s dental impression (Coltoflax, Switzerland), was used to stabilize the subject’s head to a head holder during trials. The device stabilized the head by having it rest on the upper jaw, thus avoiding the need for jaw muscle activation. The bite-board was attached to the head holder that was mounted on the chair but could be rotated independently in the horizontal plane (Fig. 1, *inset*). A torque transducer in the shaft of the head holder measured horizontal head torque (Burster, Gernsbach, Germany; range 0–20 Nm, nonlinearity <0.2% of rated output).

The chair and head holder were rotated by two independent position-controlled servomotors. Angular displacements were measured using potentiometers connected to axis of the chair and head motors. By driving the two servomotors independently, three types of slow rotational stimulation could be delivered: *1*) Head-on-trunk (“head-only”) kept the trunk stationary and evoked a combined neck and vestibular stimulation (Fig. 1). *2*) Trunk-plus-head (“whole body”) imposed a vestibular-only stimulation. *3*) Trunk-under-head (“trunk-only”) kept the head stationary and evoked a neck-only proprioceptive stimulation. Slow head rotation profiles were smoothed trapezoids with durations of 5 s and a peak displacement of 16° either to the left or to the right with a peak angular velocity of 5°/s (Fig. 1). Head-on-trunk torsions were thus restricted to the C1–C2 segments and were well within the physiological range of neutral zone (17). Importantly, these relatively small displacements did not produce any discomfort.

**Figure 2.**
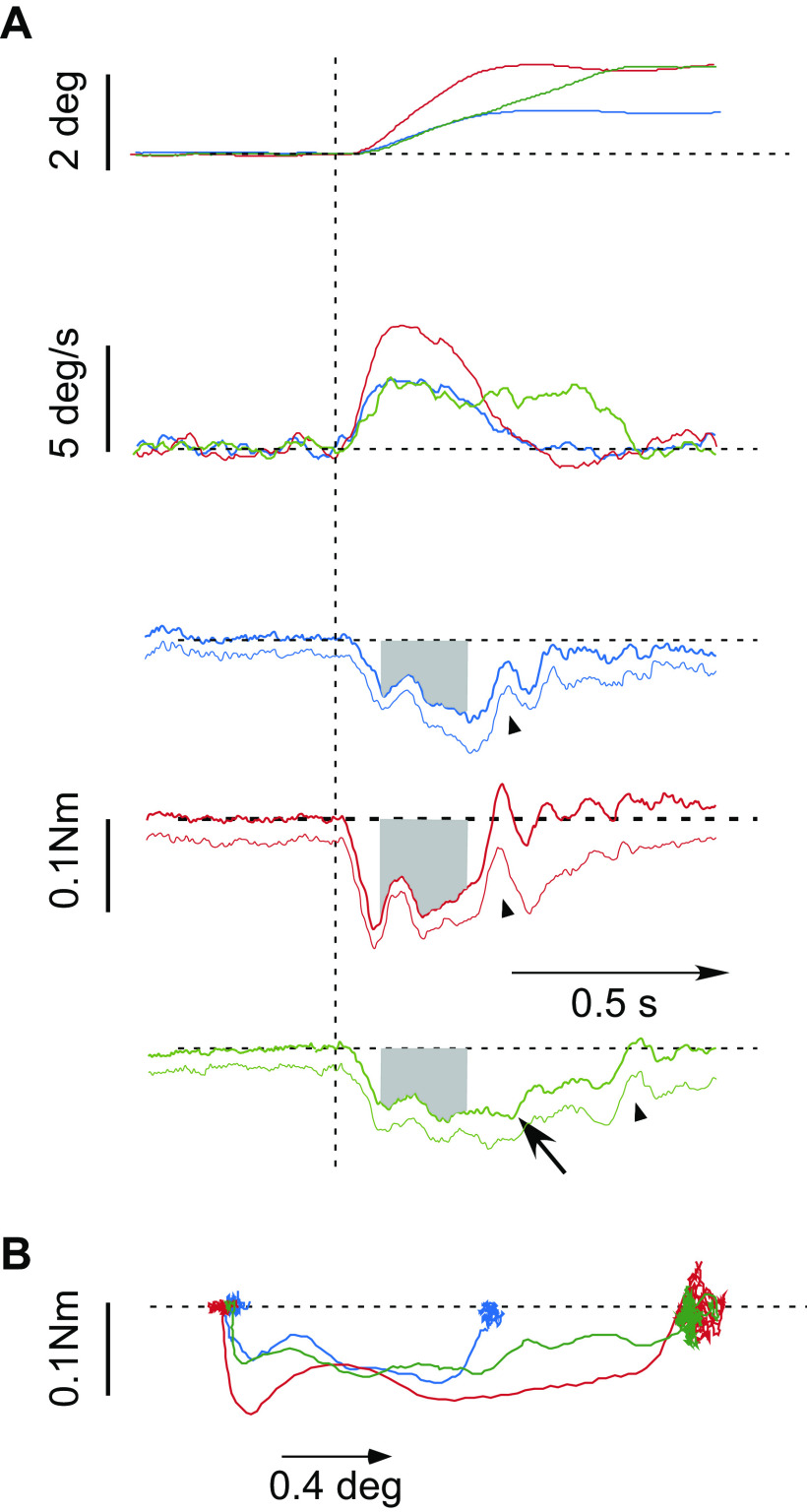
*A*: development of average resistance to brief standard (blue), fast (red), and long (green) ramp-and-hold displacement perturbations delivered at rest with the head held stationary (3rd, 4th, and 5th records from the *top*). Variation of resistance across the 6 subjects is indicated by thin lines showing average torque minus 1 SD. The calibration applies to all records. The 2 *top* panels present corresponding color-coded traces of perturbation average displacement (*top*) and velocity (second from the *top*). Perturbation onset is shown by the vertical dashed line. Note the early rise and fall of resistive torque and its reversal during the displacement deceleration phase (arrowheads). Resistance has completely subsided as soon as the head is again stationary on the trunk after displacement end. Resistance upon the 0.7-s long perturbation begins, however, to decrease gradually before stimulus end at approximately 300 ms after stimulus onset (oblique arrow). The stretch response was quantified as the torque integral separately over the time up to 100 ms (interval t_1_) and between 101 und 300 ms after stimulus onset (interval t_2,_ time integral as gray areas). *B*: torque-angular displacement diagram of the average responses shown in *A*. The slope of curves equals the overall stiffness of the head-neck system to the brief, unpredictable displacements. Stiffness increases rapidly at stimulus onset. Compliance (the reverse of stiffness) increases gradually even before the termination of the long perturbation (green curve), i.e., after about 300 ms as the head has moved by approximately 1°, indicating that the increase of compliance is time dependent, not displacement dependent.

Either during the progression of the head-only 16°-5 s slow rotation (∼2.5 s after the onset) or in isolation (sitting motionless at rest, the head held in primary, “resting,” position), a brief ramp-and-hold perturbation of head-on-trunk displacement was randomly delivered (“dynamic” or “static,” respectively). When imposed in isolation as the head was held stationary in the resting position (static), the perturbation was followed after 1 s by a distracting whole body rotation. Three different metrics of the perturbing ramp-and-hold displacement excursions resulted from short velocity pulses fed into the head motor taking one of the two different values of amplitude/duration: *1*) 0.9° (±0.01 SD) with a duration of ∼0.4 s (“standard,” blue traces, Fig. 2*A*), *2*) 1.8° (±0.02 SD) with a duration of 0.4 s (“fast,” red traces), and *3*) 1.8° (±0.01 SD) with a duration of 0.65 s (“long,” green traces). After a short acceleration time of ∼90 ms, perturbation velocity attained a plateau of ∼3, 6, and 3°/s, respectively, before the deceleration begun at ∼0.3, 0.3, and 0.6 s, respectively, after perturbation onset.

Although the implementation of 16°-5 s trunk-under-head slow rotations (resulting in isolated neck proprioceptive stimulation) was possible by adjusting the signals driving the servomotors to the chair and head holder mechanical properties, the realization of 0.9°-0.4 s trunk-only ramp-and-hold perturbations proved technically impossible to achieve with the available apparatus. Thus, trunk-under-head rotations served only as distractors (see *Protocol*).

Due to the head holder/bite-board arrangement and depending on anatomical head and upper cervical column features of each subject, the rotation axis passed a few centimeters behind the intersection of the interaural and naso-occipital lines. The head was aligned with the midsagittal plane of the trunk before testing. Care was taken to avoid discomfort in subjects during the measurements.

### Protocol

**Figure 3.**
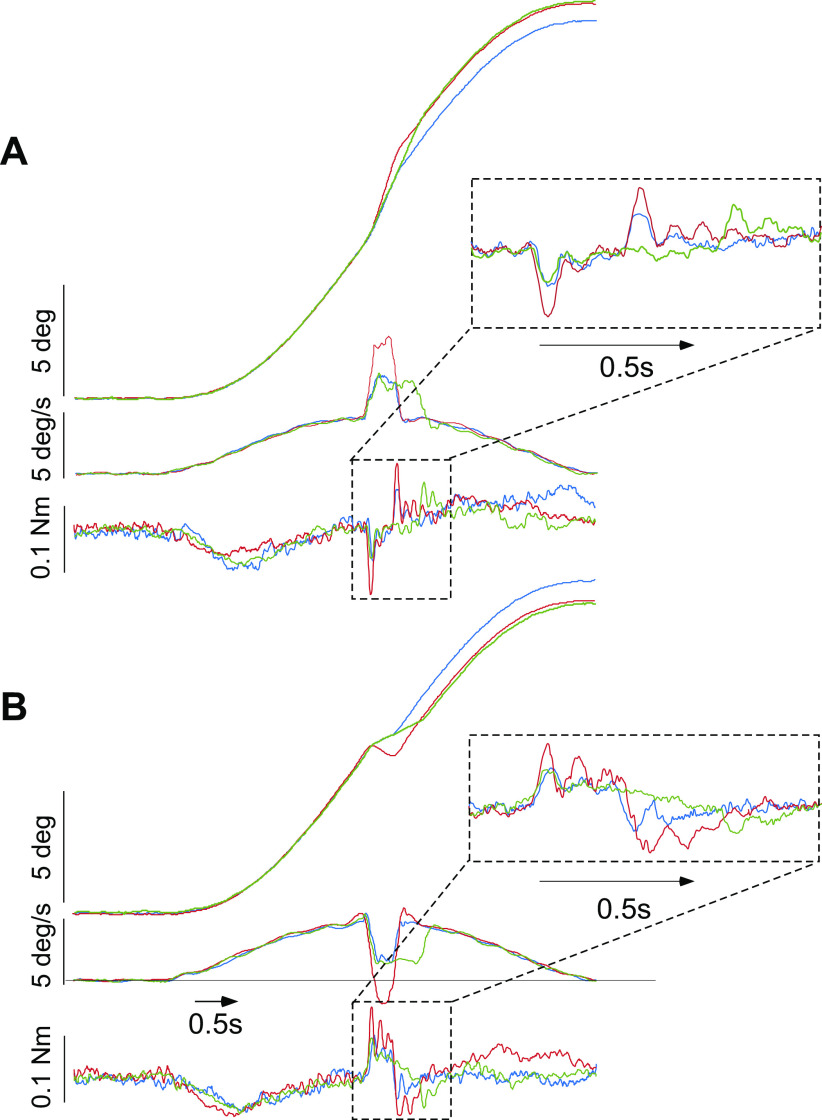
*A*: average resistance to the standard (blue), fast (red), and long (green) perturbations as in Fig. 2 when, in this case, they are delivered unexpectedly in the middle of the on-going raised-cosine head-only rotations; at this time, the velocity of externally generated head-on-trunk rotation was approximately 5∘/s and resistance to movement had dropped to 0. The perturbations moved the head further into the direction of the on-going rotation (ipsilateral). Note that resistive torque, although weaker as compared with that elicited at rest in Fig. 2 is not completely eliminated, suggesting that the gain of stretch reflexes had not been previously set to 0. *B*: average resistance when the delivered perturbations are directed opposite to the on-going raised-cosine head-only rotations (contralateral). Head-on-trunk velocity was reduced or even reversed. Note again, that resistive torque, is weaker as compared with that elicited at rest (shown in Fig. 2) but stronger as compared with that elicited upon ipsilateral perturbations. *Insets*: time base and amplification scale are given as in Fig. 2 to facilitate comparisons with the torque generated upon the perturbations delivered when the head is held stationary. Torque calibration applies to all sets of records.

Our experimental design aimed at extracting parameters of the hypothesized suppression mechanism, such as delays, thresholds, and gains of the sensory modalities/motor commands involved by comparing stretch reflexes evoked by the brief ramp-and-hold perturbations of head-on-trunk position (velocity pulses) presented unexpectedly either at rest or during the progression of a slow passive head-on-trunk rotation. Testing took place in darkness, and environmental noise was reduced through earplugs. During a session lasting ∼40 min, 32 slow 16°-5 s rotational stimuli (4 whole body, 4 trunk-only, and 8 head-only × 2 directions, leftward and rightward) were presented in random order set by the MATLAB command *randperm*. Rest periods of a half minute were given between rotations to prevent aftereffects. Twenty rotations served as distractors (2 whole body, 4 trunk-only, and 4 head-only × 2 directions), whereas 12 of them (2 whole body and 4 head-only × 2 directions) were delivered in combination with a ramp-and-hold perturbation. The perturbations were interjected randomly either when the head was held stationary (at rest), shortly before the application of the whole body distracting rotation (static perturbation, Fig. 2*A*) or in midcourse of the progression of the head-only rotation (dynamic perturbation, Fig. 3). At this time, that is, 2.5 s after the rotation onset, ∼8° of slow head-trunk displacement had been covered, the head movement, externally generated by the head holder, had attained 5°/s, and resistive torque had completely subsided and dropped to zero. When delivered during the progression of the slow passive head-on-trunk rotation, the perturbation was directed either further into the direction of the on-going rotation (“ipsilateral,” Fig. 3*A*) or opposite to it (“contralateral,” Fig. 3*B*). Thus, resistance to a perturbation was tested twice leftward and twice rightward under each of the three different context conditions (static/dynamic ipsilateral/dynamic contralateral). Each subject completed three separate sessions, one for each of the different perturbation metrics type (standard, fast, and long) with an interval of 3–5 days between them. The same six subjects were tested throughout the measurements.

Spatial attention of the subjects was attracted at their “subjective straight ahead” direction before and after each rotation (15). They were instructed to relax their neck muscles and to not resist the rotation.

The random distribution of trials containing the brief ramp-and-hold displacements among distracting head-only, trunk-only, and whole body 16°-5 s rotations in rapid succession and the engagement of attention to the “straight ahead” task ensured the unpredictability of perturbations. Asked at the end of sessions, all subjects confirmed that the weak vestibular/proprioceptive/acoustic stimulation was not subjectively startling and that they were unable to model the rotational stimuli/perturbations. Learning, anticipatory, or habituation effects were thus by and large eliminated.

### Data Acquisition and Analysis

Angular displacement and reactive head torque were sampled at 200 Hz for off-line analysis. Thus, the responses of a subject to a particular perturbation in a certain context (static/ipsilateral dynamic/contralateral dynamic) and of certain metrics (standard/fast/long) consisted of the average of two leftward and the average of two rightward responses. Resistive torque was quantified by numerically integrating in MATLAB the response separately over *1*) the first 100 ms (t_1_) and *2*) the time interval between 101 and 300 ms after stimulus onset (t_2_, gray surfaces in Fig. 2).

Notably, Ag–AgCl disk electrodes (Kendall ARBO) were attached over the belly of sternocleidomastoid muscles in five subjects, and EMG signals were amplified and analog filtered with a passband of 32 Hz to 3.2 kHz. As no bioelectrical activity could be recorded either as background or in conjunction with the rotations/perturbations throughout measurements, it was concluded that subjects were essentially trying to comply with the instruction to relax and, in addition, that the weak reflexive/voluntary torque responses to the slow speed displacements have stemmed from not directly accessible, deeper neck muscles. The trials (<10%) with large torque fluctuations (exceeding the mean ± 1.5 SD) and inadvertent EMG activation before the application of the perturbation were discarded.

### Modeling

**Figure 4.**
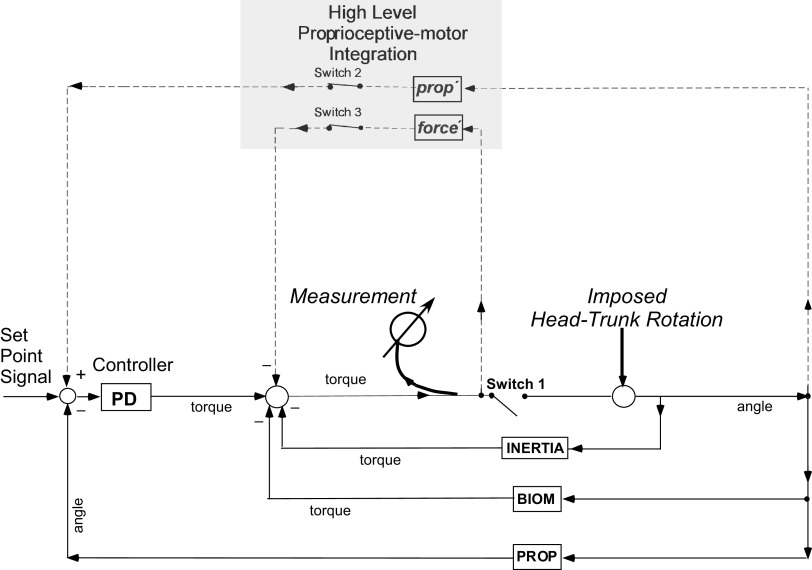
Dynamic control model of reflexive head stabilization based on earlier experimental work (15) with additional assumptions resulted from the present measurements. The *bottom* part of the drawing represents schematically the local (segmental) stretch reflex, whereas the *top* part represents the proposed mechanism of voluntary suppression. Because the head is securely fixed to the rotation device, the torque generated by neural and passive elements does not evoke any head movements (**Switch 1** is open). Head-on-trunk movements are imposed externally through the device. Voluntary suppression of the reflexive and passive viscous-elastic resistance elements is implemented via 2 feedback loops (dashed lines) conveying length and force information when *switches 2* and *3*, respectively, are (voluntarily) closed. When voluntary movements are to be executed, *switches 2* and *3* are open and **Switch 1** is closed. Dashed lines and italics are used for the hypothesized mechanism of suppression. angle, Angular displacement in yaw; PD, proportional-derivative.

For simulations, MATLAB/SIMULINK (The MathWorks Inc., Natick, MA) was used. Fits were based on the previously presented model of head control derived from older normal subjects and parkinsonian patients (15). The model describes the signal flow from passive head-on-trunk displacements to the generated forces acting on the head. The head is thereby firmly attached to the head holder, which is connected to the position-controlled head motor. The head-neck system was divided into several interacting subsystems each considering a simple physiological mechanism (18, 19). According to this approach, passive head-on-trunk rotations include system mechanics, neck reflexes, and voluntary effects (Fig. 4). Neck muscles, tendons, and fasciae exert passive elastic forces proportional to head-trunk displacement and viscous forces proportional to the velocity of head-trunk displacement (labeled jointly **BIOM**) acting against head-trunk rotations. They are given, therefore, minus sign in the model. The inertial force, due to the head mass, is proportional to the acceleration of head-trunk displacement and is also given a minus sign. Reflexively generated forces arise from vestibular and proprioceptive signals responding to head motion in space and muscle lengthening, respectively. They activate the motoneuronal pool in the cervical spinal cord (**PD Controller**). Due to the low head-to-trunk velocities used in the protocol of the earlier measurements, the recorded vestibularly generated resistance to head-on-trunk rotations was negligible and has been, therefore, for simplicity omitted (see discussion). The proprioceptive negative feedback loop (labeled **PROP**) feeding the PD controller opposes head-trunk rotations and is given also a minus sign. The reflexive torque sums with the torques generated by the passive elastic, viscous, and inertial forces to produce a net torque that is detected by the torque transducer in the shaft of the head holder. The net torque is prevented from moving the head, because the latter is firmly attached to the head holder and can rotate upon the stationary trunk only upon computer generated signals sent to the head motor. **Switch 1** is, therefore, open, that is, the model operates in an open-loop arrangement. The forces generated by system mechanics and neck reflexes have been shown to interact linearly in cats (18). Admittedly, nonlinearities can be, however, not excluded when the system is investigated in humans under a variety of conditions. When subjects are asked to relax, stretch reflexes have been thought to be suppressed through a process of neutralization instead of being directly attenuated by a descending supraspinal signal. In our previous work, neutralization has been implemented by feeding the displacement signal with a positive sign (“**prop′**,” dashed line), that is, it diminishes the torque produced by the local main proprioceptive loop **PROP** (“set point servoing”). Estimates of model parameters in the present study were calculated by optimizing curve fits (*F*) to average experimental torque responses (*aE*) upon the three static perturbations by means of a constrained numerical function (*fmincon*). The total error corresponding to a set of model parameters, common to the three fitted curves, was derived from the sum of the cumulative absolute errors of each of them (i.e., $\sum_{i=1}^{i=3}\left\{\int_{t=1}^{t=650\;\text{ms}}[tF-aE]\cdot \text{d}t\right\}$, counted over a time interval of 650 ms after perturbation onset, where *tF* denotes the measured torque value and *aE* the torque value predicted by the numerical simulation of the model). The unconstrained parameters to be identified were allowed to vary within a certain, “physiological,” range provided in the literature (cf. 16, 19, 20). Iterative simulations were carried out aiming at minimizing the total error. The parameters converged toward single values.

### Statistics

As multiple correlated measurements were made on each of the six subjects, a linear mixed-effects analysis was used to outline the relationship between the dependent variable of interest **Y** (i.e., resistive torque) and the various perturbation metrics (standard/fast/long), context (static/ipsilateral dynamic/contralateral dynamic), or direction (left vs. right; 21). Random intercept models were used with intercepts for subjects being entered as random effects (*u*). Perturbation metrics, context, or direction were introduced into the models as fixed effects (**β**) with or without interaction terms.

In matrix notation:

Y=Xβ+Zu+ε,

where **Y** is the *n* × 1 vector of responses, **X** is a *n* × *p* design/covariate matrix relating the responses to the fixed effects **β**, and **Ζ** is the *n* × *q* design/covariate matrix relating the responses to the random effects *u* (Stata SE 14.1, StataCorp LP). The *n* × 1 vector of errors ε is assumed to be multivariate normal with mean 0 and variance matrix ${\text{σ}}_{\text{ε}}^{2}R$. Chi-square values were rescaled to *F* ratios. Likelihood ratio (*lr*) tests yielded additional proof of significance by comparing the full model with the effect of a fixed term (i.e., perturbation metrics or context) against the reduced model without the effect of the same term. Significant difference between the likelihood of these two models would confirm that the fixed term under consideration affects the dependent variable **Y** (i.e., resistive torque). Significance levels obtained by means of the two approaches were always congruent (Table 1). The threshold for rejecting the null hypothesis was set at *P* < 0.005 in all tests. Deviations from homoscedasticity were estimated by visual inspection of residual versus fitted values plots. Data are generally reported as means ± 1 standard deviation (SD).

**Table 1.** Comparisons between the effects of perturbation metrics and behavioral context on resistive torque

Perturbation Context	Perturbation Metrics	Standard	Fast	Long	Significance of Fixed Effects
*Early Response (up to 100 ms after perturbation onset)*					
Static		3.9 ± 1.5	7.5 ± 1.5	3.5 ± 1.0	*1*) Perturbation metrics (standard/fast/long): *F*_2,86_ = 81.2, *P* < 0.00001 χ^2^ = 94.9, *P* < 0.00001 *2*) Perturbation context (static/ipsilateral dynamic/contralateral dynamic): *F*_2,86_ = 11.4, *P* = 0.00004 χ^2^ = 20.2, *P* < 0.00001
Ipsilateral dynamic		2.6 ± 1.0	6.8 ± 1.3	3.4 ± 0.6	
Contralateral dynamic		2.4 ± 1.3	4.8 ± 1.2	3.2 ± 1.0	
*Late Response (between 101 and 300 ms after perturbation onset)*					
Static		13.4 ± 5.1	16.4 ± 7.5	11.2 ± 3.8	*1*) Perturbation metrics: *F*_2,85_ = 2.8, *P* = 0.06 χ^2^ = 5.5, *P* = 0.06 *2*) Perturbation context: *F*_2,85_ = 45.7, *P* < 0.00001 χ^2^ = 64.1, *P* < 0.00001
Ipsilateral dynamic		3.8 ± 2.2	3.4 ± 4.5	2.7 ± 2.8	
Contralateral dynamic		5.6 ± 5.5	7.3 ± 5.3	4.8 ± 5.0	

Integral of the early (over the first 100 ms after stimulus onset) and late (between 101 and 300 ms) resistive torque to the ramp-and-hold perturbations in Nm*s*10^−3^ (mean values ± 1 SD across 6 subjects). The size of the early response is graded mainly with the rate of stretch and to a lesser degree with the perturbation context, whereas the late response is graded only with the perturbation context. The effect of direction (left vs. right) and interactions between the effects perturbation metrics and context were nonsignificant.

## RESULTS

Torque responses of healthy subjects upon low-velocity, smoothed trapezoidal head rotations have been reported, modeled, and illustrated in earlier publications (14, 15). In short, normal subjects exert negligible force on the bite-board at rest. When the neck is deflected on the stationary trunk while subjects have been asked to relax, resistive torque rises with an initial, relatively steep slope, before leveling off and later decreasing within several hundreds of ms (Fig. 1, representative raw data, *top* records; mean torque and variation across subjects, *bottom* records). It can at times even reverse. Substantial reflexive responses to low-velocity, smoothed trapezoidal, whole body rotations (i.e., vestibular stimulus of up to 18°/s) are missing. Here, we concentrate on the responses upon the three different ramp-and-hold perturbations applied when the head was held stationary (static perturbations, Fig. 2) and compare differences when these were superimposed in midcourse of the slow, passive head-on-trunk rotations (dynamic perturbations, Fig. 3).

### Responses upon Static Perturbations

When the perturbations were delivered at rest (i.e., when the head was held stationary), torque rose initially rapidly before leveling off during the remainder of the stretch (Fig. 2). A similar but oppositely directed fast resistance variation was recorded simultaneously with the deceleration phase of the perturbation (arrowheads), followed by almost complete relaxation as soon as the head was again stationary on the trunk. This transient fluctuation of resistance at perturbation onset/termination was recorded stereotypically upon all the three different perturbations. The initial torque rise represents presumably the sum of resistance to head movement from several sources, such as inertial force, passive stiffness including short-range stiffness (detachment of cross bridges), and short-latency cervicocollic reflex responses. The size of this early response, quantified over the first 100 ms after perturbation onset, is graded with the rate of stretch (Table 1). During the following 200 ms (i.e., between 101 and 300 ms after stimulus onset), the level of the late reflexive response remained more or less constant (Fig. 2). Clear torque level reductions before stimulus end were recorded only upon the 0.7-s long perturbation, that is, they began not earlier than at ∼300 ms after stimulus onset (Fig. 2, oblique arrow).

### Responses upon Dynamic Perturbations

When the perturbations were superimposed unexpectedly during the on-going head-trunk passive displacements (Fig. 3, *A* and *B*), resistance during the initial time interval of 100 ms after stimulus onset was by ∼20% on average reduced, as compared with that recorded when they were delivered at rest (compare mean values ± SD in Table 1). During the interval between 101 and 300 ms after stimulus onset, “dynamic ipsilateral” responses were considerably reduced, amounting to between 20% and 30% of the static responses, suggesting that stretch reflexes were not completely turned off (Table 1). Furthermore, also the “dynamic contralateral” late responses after stimulus onset were weaker than the static responses (amounting to ∼40% of their size) indicating that the instruction to relax modulated the stretch responsiveness of both antagonists and agonists neck muscles during the passive head-on-trunk movement. Torque oscillated several times at ∼9 Hz before coming to the preperturbation low level.

### Simulations

**Figure 5.**
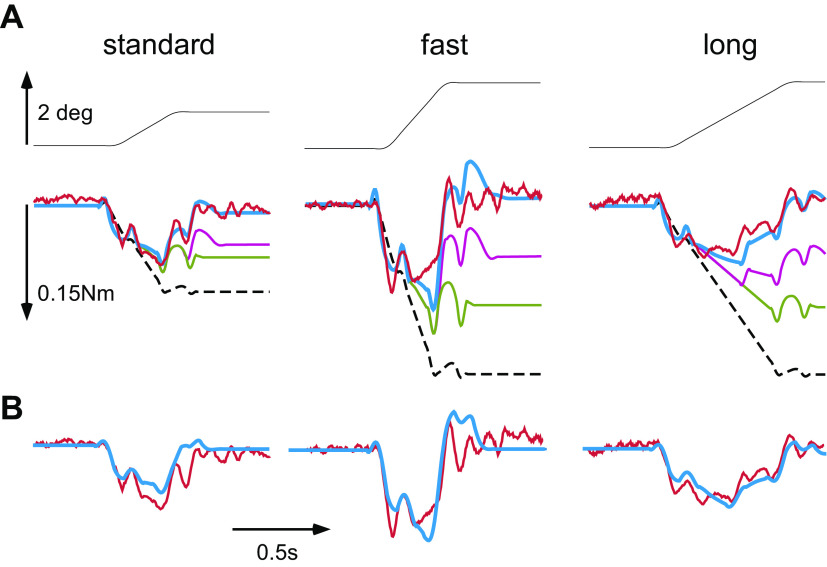
*A*: simulated torque-time traces (blue) upon the static perturbations predicted by the model in Fig. 4 (controller*:* proportional factor 0.09 Nm/°, derivative factor 0.007 Nm*s/°, delay time 50 ms; passive elements: stiffness 0.008 Nm/°, damping 0.002 Nm*s/°, inertia 0.048 kg·m^2^, no delay time, damping ratio as a whole 0.51). Experimental responses are superimposed in red. Predicted traces after the successive removal of force and displacement “neutralizing” feedback are depicted in magenta and green, respectively. Dashed traces represent predictions when using the parameters estimated by Tangorra et al. (22), i.e., an overall stiffness factor of 0.12 Nm/°, a damping factor of 0.006 Nm*s/°, and an inertial factor of 0.018 kg·m^2^. *B*: 3-fold cross-validation of the model. The resistance upon 2 out of the 3 perturbations with the head held stationary was used as the training set of data to fit model parameters. Subsequently, the learned parameters were used to predict the response upon the remaining third perturbation (validation set), and performance of the model was assessed by comparing the predicted (blue traces) with the experimentally obtained resistance upon each 1 of the 3 static perturbation (red traces). Training and validation sets crossed over in 3 successive iterations.

Responses upon the low-velocity, smoothed trapezoidal rotations reproduced data and model parameters obtained from normal subjects which have been published earlier (15). Fits of the responses upon the three static ramp perturbations with the previously presented model indicated, however, that additional assumptions are needed to explain the gradual torque level reduction ∼300 ms after stimulus onset and the almost complete relaxation achieved immediately after the termination of perturbations. Resistance reductions were now implemented by *1*) the previously hypothesized neutralizing length tracking mechanism ***prop*′** and, in addition, by *2*) feedback loops signaling contact forces. Force feedback is here thought to reduce not only the output of the actuator **PD** but also the resistance due to the passive muscle and tendon elements **BIOM** (“contact force rejection,” cf. discussion). For simplicity, a single loop (***force*′**) was used in simulations originating from the net production of torque and reducing it after running through a low threshold, a delay, and a gain factor (dashed loops in Fig. 4). With this additional assumption, a good approximation of the experimentally derived average torque traces was now possible (Fig. 5*A*).

Relevant model parameters included the following:

Controller: proportional factor 0.09 Nm/°, derivative factor 0.007 Nm*s/°, delay time 50 ms (lumped value for all delays in the control loop; cf. 16).

Passive elements (**BIOM**): stiffness 0.008 Nm/°, damping 0.002 Nm*s/°, no delay time.

Head moment of inertia: 0.018 kg·m^2^ (cf. 20). An additional acceleration-dependent factor, amounting to 0.030 kg·m^2^ was introduced to explain adequately the magnitude of the stereotypical initial sharp rise and fall of torque and the oppositely directed resistance variation during the deceleration phase of head-trunk displacement. This additional acceleration-dependent element has to be attributed to the cantilevered carriage of the head on the cervical vertebral column, the eccentric position of the head mass in relation to the rotation axis, in which the torque gauge was positioned. When the rotation axis of a sphere is eccentric, the moment of inertia increases to

$$J=\frac{2}{5}\cdot m\cdot r_{0}^{\text{2}}+m\cdot r^{\text{2}},$$

where *r*_0_ is the radius of an ideal sphere of mass *m* and *r* is the distance of the rotation axis from the center of the sphere (cf. discussion).

The estimated value of the damping ratio of the proposed configuration as a whole equals to 0.51 (neglecting latency), indicating that the head-neck system, as modeled by means of our experimental data, responds in an underdamped fashion.

Voluntary suppression of reflexive head stabilization via length feedback (***prop*′**, *switch 2* closed): Displacement threshold 0.6°, delay time 70 ms, gain 0.9.

Voluntary suppression of reflexive head stabilization via force feedback (***force*′**, *switch 3* closed): Threshold 0.02 Nm, delay time 90 ms, gain factor 4 s^−1^ (Laplace transform).

Compared with the experimental traces, predictions were grossly erroneous if torque reduction was implemented solely through inhibitory force feedback, that is, after completely removing the neutralizing length tracking loop ***prop*′** (Fig. 5). Simulations by simply reducing the gain of the reflex pathway **PROP** below 1, that is, by means of gain control (gain scheduling), yielded larger errors than those estimated by means of the proposed neutralization via length and force feedback. As a matter of fact, gain scheduling predicts complete relaxation for the period after the termination of stretch only under the unrealistic assumption of zero stiffness of the passive elements (**BIOM**).

A threefold cross-validation of the model has been performed by using the resistance upon two out of the three perturbations with the head held stationary as the training set of data to fit model parameters. Subsequently, the “learned” parameters thereof were used to predict the response upon the remaining third perturbation and performance of the model was assessed by comparing the predicted with the experimentally obtained resistance (Table 2). Training and validation sets crossed over in three successive rounds (3-fold cross-validation). The results are given in Table 2. Simulated torque-time traces (blue) predicted by the parameters from the training set are superimposed upon the experimentally derived resistance (red) for each of the “validated” perturbations in Fig. 5*B*.

**Table 2.** Threefold cross-validation of the model

Training Set	Average Error When Fitted to the Training Set	Validation Data	Prediction Error
Torque responses		Torque response	
Upon the standard and fast perturbations	21%	Upon the long perturbation	22%
Upon the standard and long perturbations	19%	Upon the fast perturbation	25%
Upon the fast and long perturbations	18%	Upon the standard perturbation	34%

The resistance upon 2 out of the 3 perturbations with the head held stationary was used as the training set of data to fit model parameters. Subsequently, these learned parameters were used to predict the response upon the remaining third perturbation (validation set) and performance of the model was assessed by comparing the predicted with the experimentally obtained resistance (prediction error). Training and validation sets crossed over in three successive iterations. The error of the algorithm when fitted to the training set was computed as the average of the cumulative absolute errors of each of the two fitted curves counted from the onset until perturbation end. Similarly, the prediction error of the fitted model was estimated by predicting the validation data from the onset until perturbation end. To facilitate comparisons and interpretation, error is given in the table as percentage of the integral of the experimentally derived average torque responses until perturbation end. A graphic representation of the prediction error is illustrated in Fig. 5*B*.

**Figure 6.**
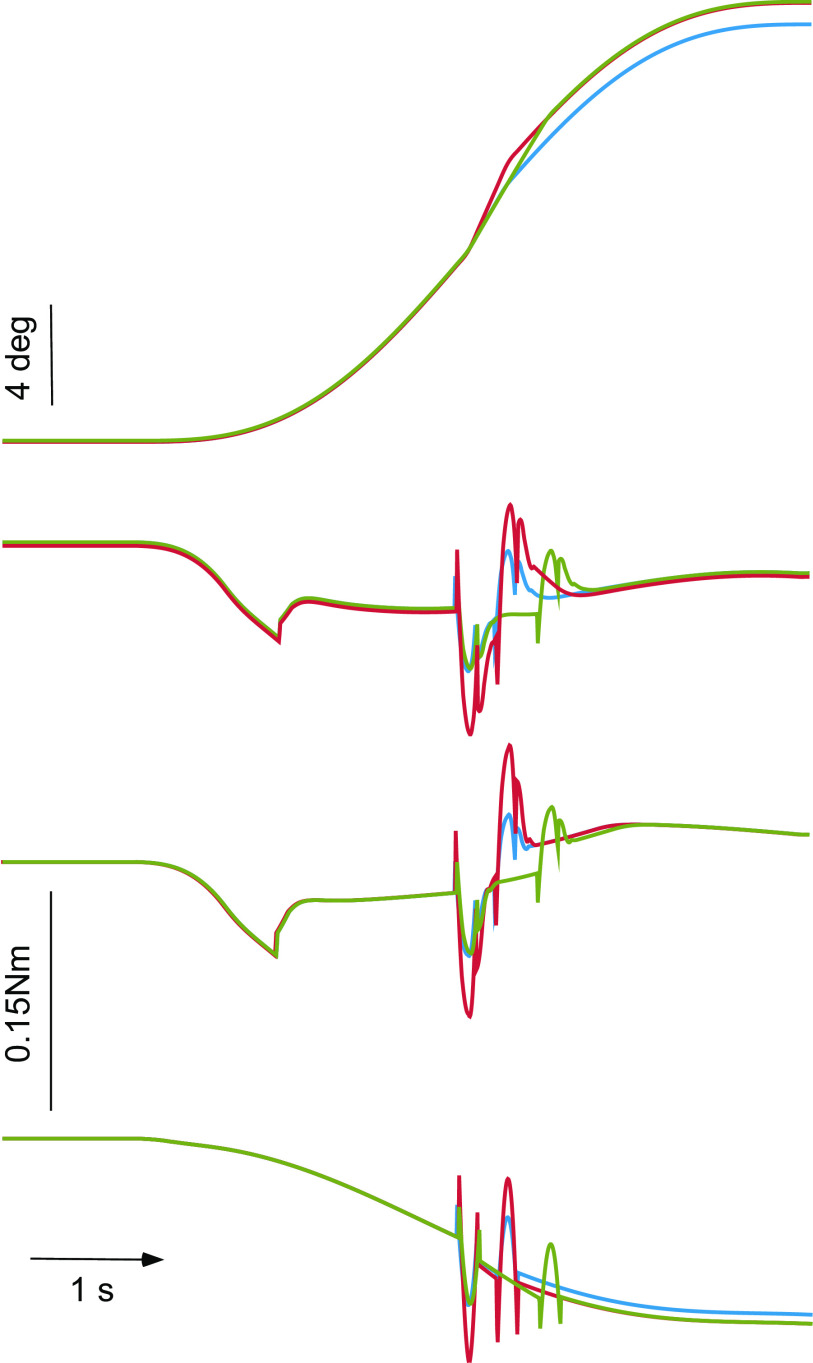
Simulated traces to the standard (blue), fast (red), and long (green) perturbations as in Fig. 2 when they are delivered unexpectedly in the middle of the on-going raised-cosine head-only rotations (*top*). The experimentally derived resistance is illustrated in Fig. 3*A*. The calibration applies to all records. The *second* panel from the *top* represents resistance predicted by the model in Fig. 4 with parameters estimated from fitting the 3 static perturbations. The *third* panel shows predicted resistance after reducing the delay of length neutralization loop (***prop*′**) by 20 ms and increasing gain by 0.2, whereas the *fourth* panel depicts resistance predicted by gain scheduling.

When the above estimated values were used to predict resistance upon perturbations superimposed in midcourse of the on-going passive head-trunk rotations, fits were on average 20% (early response) and 100% (late response) larger than the experimentally derived resistive torque (Fig. 6). Although the predicted late response could be reduced down to the level of the experimentally derived one by slightly manipulating the delay time and gain of the suppressive length feedback loop (reduced down to 50 ms and increased up to 1.1, respectively), the early response remained unaffected by these modifications.

Given the attainment of complete relaxation immediately before the deployment of the perturbation, the candidate model of gain control predicts the absence of any measurable, neurally mediated resistance upon the transient rise of head velocity (cf. discussion).

## DISCUSSION

In this study, we investigated the mechanism of neck reflexes suppression when the head is rotated passively on the stationary trunk and subjects are asked to relax. By applying randomly and unexpectedly brief displacement perturbations during externally generated, passive head rotations and by precluding prediction, we demonstrated the involvement of proprioceptive feedback in suppressing neck reflexes. Furthermore, we constructed, building on previous experiments, a physiologically plausible, relatively simple neuromechanical model reproducing key features of the suppression process. According to this model, resistance to stretch generated by passive, noncontractile elements and reflexively mediated muscle contractions is voluntarily “neutralized” or “cancelled” by mirror length and force feedback loops. The proposed model of stretch reflex neutralization is based on a large number of measurements in normal subjects and patients with movement disorders. The candidate model of stretch reflex gain adjustments to zero, as an exclusive neck reflexes suppression mechanism, cannot reproduce key resistance changes to passive head displacements as they were observed in the present and previous investigations (cf. also Ref. 16). Voluntary neck muscle relaxation, similarly to muscle contractions during purposeful motor tasks, relies on length and force feedback from the periphery.

### Source of Resistance to Imposed Head-Neck Rotations

It was not the prime aim of the present experiments to identify and discriminate between the various components contributing to the stretch response of the head-neck system. Instead, we sought to infer and model the underlying neuromechanical suppressive mechanism of head-neck reflexes when humans are asked to relax. The profile, timescale, and simulations of torque development to the ramp-and-hold displacement perturbations with the head held stationary (Figs. 2 and 5) yielded, however, some clues as to the sources of resistance. We suggest that most of the velocity-dependent resistance within the first 100 ms after stimulus onset can be attributed to inertia. Noticeably, the duration of the ramp-and-hold acceleration period was ∼90 ms. Accordingly, an oppositely directed fast torque variation was recorded simultaneously with the deceleration phase of the perturbation (arrowheads, Fig. 2). The stereotypical reversal of resistance in this instance does not support the assumption of strong contributions from passive/elastic muscle mechanisms such as short-range stiffness (23, 24). Resistance attributed to short-range stiffness probably amounts to ∼20% of the early response. Indeed, the early response was reduced by 20% when the ramp-and-hold perturbations were superimposed in the midcourse of the progression of the head-only rotations, that is, when the cross bridges had been, due to the previously effected reflexive contraction, just detached and the proposed feedback mechanism had not taken effect yet. Admittedly, vestibularly evoked components within the first 100 ms cannot be completely excluded. Still, vestibulocollic effects in the horizontal plane at these low velocities and frequencies are insignificant in alert humans and primates (1, 2, 15, 25, 26). Similarly, vestibulocollic responses to natural stimulation in the horizontal plane in animal preparations are negligible, below 5°/s, even by means of intramuscular EMG recordings (27). Strong nonlinearities probably exist raising a threshold. Nevertheless, the evoked low-velocity vestibular signals, being above the perception threshold of 1–2°/s, are thought to combine with neck-proprioceptive signals in reconstructing external disturbances to be rejected according to the task (cf. first paragraph of the following section).

Notably, the initial torque rise upon the ramp-and-hold perturbations with the head held stationary (static condition, Figs. 2 and 5) reproduces results of the head-neck dynamics parametrization calculated by Tangorra et al. (22), despite the fact that their estimates were based on the reverse information flow compared with ours (i.e., taking into account head displacements upon torque perturbations). For comparisons, we took a mean overall stiffness factor of 0.12 Nm/° and a mean damping factor of 0.006 Nm*s/° to represent their parametrization, that is, those estimates calculated when their subjects, in analogy to our protocol, did not exert any force to the helmet/perturber before the application of the torque perturbations. As the rotation axis in their study was placed nearer to the center of the head mass, the estimated level of the inertial element was correspondingly lower in their report.

In conclusion, the rapid torque rise during the first 100 ms after stimulus onset has to be mostly attributed to inertia and secondarily to passive elastic/viscous elements (among them short-range stiffness) and short-latency cervicocollic reflexes (latency ∼50 ms; 16).

The late response between 101 and 300 ms after stimulus onset has to be attributed to passive muscle/connective tissue elements and to the activation of the cervicocolic reflex, representing the major fraction of resistance to stretch during this time interval. The reflexive, “automatic” nature of this late response cannot be easily disputed, as the subjects, sincerely trying to comply with the task to relax, could not suppress it by the effort of will. Coactivation of agonist and antagonist muscles, may have contributed here, just as during the first 100 ms after displacement onset. This is, however, rather unlikely because, under the instruction not to resist, any bioelectric activity in the lengthening neck muscles ceases within ∼80 ms after the onset of stretch (16). As the latency of (voluntary) bioelectric activation of neck muscles upon somatosensory stimulation in reaction time tasks amounts to ∼100 ms (28), voluntary interference has to be accepted. Most probably, the voluntary response interacts with reflexive forces in this case and, because of the given task not to oppose, reduces them (disturbance rejection). The late response may be thus thought as a kind of context-dependent “long-loop” reflex, as it was clearly modified when subjects had been given the time to adjust the suppressive mechanism correspondingly in midcourse of the progression of the head-only, 5 s passive rotation (Fig. 3 and Table 1).

### Voluntary Suppression of Resistance to Imposed Head-Neck Rotations

Stretch reflex transmission in humans can be regulated dependent on the context and instruction to match the requirements of the task at hand. For example, interaction with rigid environments or the instruction to relax decreases responsiveness, particularly of its later components (6, 29, 30). These changes might be affected, for example, through direct descending actions upon α and γ motoneurons, presynaptic terminals, or interneurons. Theoretically, reflex responsiveness could be modulated simply by changing the reflex gain (gain scheduling; 12, 31). Instead, we have earlier proposed as underlying mechanism of head-neck stretch reflexes modulation, the change of the motor servo reference by subtracting from its input, in the simplest model version, ongoing sensory signals from the periphery (14, 15, 32). To this end, physical variables of external disturbances having impact on the body are online reconstructed through the integration of impinging sensory signals. For example, the perception of head-trunk displacement through isolated vestibular or neck-proprioceptive stimulation (i.e., whole body or trunk under the stationary head rotations) is erroneous/illusory. Veridical head motion perception is dependent upon the combination of the two modalities during head-on-trunk rotations (33). After this intermediate level of processing, the estimate of the reconstructed physical variable is “injected” into the local proprioceptive loop to reject the external disturbance according to the task at hand. This mechanism has explained several findings in our earlier work such as torque reversals in control subjects and the fact that abnormal resistance of parkinsonian and dystonic patients was more prominent during low-velocity passive head-on-trunk rotations. Simulations of the present experimental findings suggested now that not only displacement (length) but also force signals may be involved in changing the motor servo reference. In the flow diagram of Fig. 4, head-neck angle information (gained through intersensory processing of neck and vestibular signals, ***prop*′**) is directly fed off to the motor servo of lengthening muscles with a positive sign, that is, it diminishes the torque produced by the local main proprioceptive loop **PROP**. Force signals fed from the periphery may reduce resistance both by inhibiting the motor servo of the lengthening muscles and, as proposed here, by activating the motor servo of the shortening muscles (***force*′**). Force signals, stemming according to the proposed scheme not only from Golgi tendon organs but also from joint receptors and pressure receptors on the teeth, combine to reconstruct the physical variable of the external contact force. The activation of shortening muscles simultaneously with the inhibition of lengthening muscles has been previously demonstrated experimentally in the study of Corna et al. (16, Fig. 4*Ab*). This pattern of response is not expected to result in cocontraction and, therefore, in increased stiffness. Indeed, force feedback can have both inhibitory and excitatory components in regulating the mechanical properties of large muscle groups (34). In this case, it is thought to regulate, together with length feedback, the mechanical properties of the complex multijoint head-neck system in agreement with the instructions given to the subjects. These regulatory modifications engage multiple levels of the neuraxis, probably involving sensorimotor cortical areas. In contrast, afferent information from Golgi tendon organs as part of the local, segmental control, have been recently assumed to signal the muscle force-dependent tendon length because muscle spindles by themselves cannot code for the joint position (35, 36). Thus, a veridical estimate of the joint angular position can only result by combining afferents from muscle spindles, signaling muscle length/velocity and Golgi tendon organs, signaling contracting force, provided that tendon compliance is taken into account. Along these lines of reasoning, the local force feedback is subsumed under the local main proprioceptive loop **PROP** of the model in Fig. 4. It cannot be, however, ruled out that local force feedback may exert also inhibitory effects onto the controller and that relaxation, driven by signals from the brainstem or cortex, leads in a shift in the balance of length and force feedback to favor inhibition (37).

According to the proposed conceptual scheme, the reflexive and passive torque to stretch can be suppressed or titrated in the model voluntarily, that is, the *switches 2* and *3* are thought to be operated in and out voluntarily at a cortical level (“high-level proprioceptive-motor integration” in Fig. 4). The proposed mechanism, in contrast to gain scheduling, is physiologically more advantageous, as it allows for compensation of incoming disturbances even during the suppression/relaxation time period. Indeed, after resistance had completely subsided in midcourse of the passive head-trunk displacement, it temporarily reappeared upon the superimposed ramp-and-hold perturbations (Fig. 3). By reducing the delay and by slightly increasing the gain of the suppressive length feedback loop during the slowly evolving head-trunk rotation, the proposed model of set point servoing can predict the reappearance of (weaker) resistance upon the superimposed perturbations. Such parameter modifications, enabled, for example, at a cortical level (impedance control, here admittance), are not completely unreasonable provided that enough time for adjustments has intervened. Another potential explanation for the attenuation of the response is the history-dependent reduction of the muscle spindle stiffness by constant motion. The task-dependent modulatory effects were distributed even to the stretch reflex responsiveness of antagonist, presumably contracting muscles (Fig. 3*B*). Normally, stretch responses of contracting muscles are expected to be more or less unaltered or even increased, as compared with the stationary condition (38).

In contrast, gain scheduling predicts the absence of any measurable, neurally mediated resistance. This assumption did not hold experimentally. There are further reasons to reject the hypothesis that gain scheduling is exclusively responsible for the suppression; if this were true, resistance would be expected to be steadily increasing during the deployment of the slow head-trunk rotation due to the increasing position and velocity-dependent passive forces resulting from the noncontractile tissue properties. Also, despite not being conclusive about quantitative aspects of the suppressive process, the study of Corna et al. (16) demonstrated both early (reflexive) and voluntary activation of muscles assisting the head in following the direction of the imposed force in a similar experimental setup, demonstrating that the suppressive mechanism is not simply restricted to gain adjustments of the lengthening muscles. Their study, just as the present measurements, do not support the equilibrium point hypothesis, according to which voluntary relaxation simply raises the threshold of activation of the stretch reflex outside the range of movement.

### Conclusions

We propose that voluntary relaxation of the neck muscles is, in the same way as voluntary muscle contractions, an active process relying on sensory feedback. Our results do not necessarily prove that the proposed mechanism of neutralization is the only one correct. For example, several models favor sensory reweighting and the combination with centrally generated information as a mechanism of predictive and reactive control based on state estimates derived from Kalman filters (39). Also, our results do not completely exclude interactions with “unloading” responses of the assisting muscles (40) or the additional involvement of gain scheduling control, the latter taking place particularly before the arrival of a stimulus or before the initiation of a movement/relaxation process (“anticipatory motor set” or “readiness”; 12). Remarkably, the primary motor cortex and the supplementary motor area are activated both prior and during voluntary muscle relaxation (11). Our results simply underline the involvement of sensory feedback in the sequence of events leading to the suppression of stretch reflexes during passive head-on-trunk movements. Indeed, given that the coordination of complex movement requires the temporal succession of both contraction and relaxation of the muscles involved, it is reasonable to assume that not only contraction but also relaxation is based on sensory feedback (41). Further experimental evidence is, however, needed to determine separately the time course, the relative contribution of each mechanism, and the impact of possible learning effects. Higher velocity head-trunk perturbations, evoking vestibulocollic reflexes, may be helpful in studying nonlinearities of the system and may shed light upon the issue whether distinct mechanisms are responsible for the suppression of stabilizing reflexes stemming from different modalities. Appropriately tailored physiotherapy protocols implementing enhanced sensory feedback may prove useful for the treatment of patients with movement disorder presenting with muscle tone increase (e.g., Parkinson’s disease, dystonia, Alzheimer’s disease).

## GRANTS

This work was funded by the National and Kapodistrian University of Athens (ELKE 70/3/5510).

## DISCLOSURES

No conflicts of interest, financial or otherwise, are declared by the authors.

## AUTHOR CONRIBUTIONS

D.A. and T.M. conceived and designed research; D.A. performed experiments; D.A. and L.A. analyzed data; D.A. and T.M. interpreted results of experiments; D.A. prepared figures; D.A. drafted manuscript; D.A., L.A., and T.M. edited and revised manuscript; D.A., L.A., and T.M. approved final version of manuscript.
